# Side of Onset in Parkinson’s Disease and Alterations in Religiosity: Novel Behavioral Phenotypes

**DOI:** 10.3233/BEN-2011-0282

**Published:** 2011-05-23

**Authors:** Paul M. Butler, Patrick McNamara, Raymon Durso

**Affiliations:** ^1^Department of NeurologyBoston University School of MedicineBostonMAUSA; ^2^Department of NeurologyVA Boston Healthcare SystemBostonMAUSA

**Keywords:** Religiosity, Parkinson’s disease, brain laterality, aesthetics, dopamine, frontal lobes

## Abstract

Behavioral neurologists have long been interested in changes in religiosity following circumscribed brain lesions. Advances in neuroimaging and cognitive experimental techniques have been added to these classical lesion-correlational approaches in attempt to understand changes in religiosity due to brain damage. In this paper we assess processing dynamics of religious cognition in patients with Parkinson’s disease (PD). We administered a four-condition story-based priming procedure, and then covertly probed for changes in religious belief. Story-based priming emphasized mortality salience, religious ritual, and beauty in nature (Aesthetic). In neurologically intact controls, religious belief-scores significantly increased following the Aesthetic prime condition. When comparing effects of right (RO) versus left onset (LO) in PD patients, a double-dissociation in religious belief-scores emerged based on prime condition. RO patients exhibited a significant increase in belief following the Aesthetic prime condition and LO patients significantly increased belief in the religious ritual prime condition. Results covaried with executive function measures. This suggests lateral cerebral specialization for ritual-based (left frontal) versus aesthetic-based (right frontal) religious cognition. Patient-centered individualized treatment plans should take religiosity into consideration as a complex disease-associated phenomenon connected to other clinical variables and health outcomes.

## 1. Introduction

In the 1970s, a flurry of reports ignited interest in brain-religion relationships [1–5]. These early investigators described a subgroup of patients with temporal lobe epilepsy (TLE) as expressing intense religious obsessions and preoccupations. The heightened religiosity found in the few TLE patients who reported it, however, may have been related to psychiatric effects of TLE [6,7]. In a recent exhaustive review of the literature Devinsky and Lai (2008) largely agree with Trimble and Freeman's (2006) report that *interictal* religiosity may be linked with bilateral temporal lobe seizure foci [7,8]. During *ictal* experiences however they argue that religiosity is associated with right temporal lobe activity and point out that 4 of the 5 well documented cases of 'in the moment' ictal religious experiences were associated with right temporal or temporofrontal foci. Wuerfel and colleagues (2004) evaluated religiosity and brain volumes/activity in 33 patients with partial-onset epilepsy with quantitative MRI scans [9]. Some patients had frontal lobe seizures while others had TLE. They found that religiosity in both patient groups significantly correlated with reduced right hippocampal volumes but not amygdalar volumes. Meanwhile a recent review of the literature on changes in religiosity in patients with schizophrenia and with fronto-temporal dementia also suggested a link between enhanced religiosity and right frontal temporal networks [10].

We recently reported that patients with LO Parkinson's disease (thus indicating greater right-sided disease) exhibit reduced religiosity and reduced automatic

ISSN 0953-4180/11/$27.50 © 2011 – IOS Press and the authors. All rights reserved access to religious concepts relative to RO patients [11]. In a study with 25 mid-stage PD patients compared to 25 neurologically intact subjects, patients with PD scored significantly lower in self-report measures of religiosity in addition to exhibiting deficits in priming to religious versus control concepts. Alterations in religiosity were greatest in LO versus RO PD patients. In the present study we sought to replicate and extend this previous work. We utilized a story-based priming procedure to probe alterations in religiosity in LO *versus* RO patients with PD. Recent empirical work by Norenzayan and Hansen (2006) emphasizes the importance of mortality salience in modulating religious belief [12]. These investigators showed that mortality salience increased belief in supernatural agency in healthy young adults from predominately Christian backgrounds. This work is an extension of a long tradition of theory that religion primarily functions to allay anxiety in the face of death [13,14]. Norenzayan and Hansen (2006) connected this line of historical thought with terror management theory, which predicts that human awareness of death invokes engagement of psychological buffer systems predominately reliant on culture [15,16]. To assess alterations in religiosity, we adapted the priming procedure of Norenzayan and Hansen (2006) in addition to adding a new condition: salience of beauty in nature. We include this condition because rich philosophical and theological historical traditions recurrently affirm the relationship between aesthetic sensitivity and belief in the divine, and phenomenological study of religious experience commonly implicates aesthetics in mystical, divine, or transcendent experiences [17–21].

## 2. Methods

### *2.1. Participants*

#### *2.1.1. PD Patients*

Twenty-four PD patients (2 females) were recruited from the outpatient Movement Disorders Clinic at the VA Boston Healthcare System, Boston, MA. Patients were individually diagnosed by Dr. Raymon Durso, an expert neurologist in PD, and completely fulfilled the UK PDS Brain Bank diagnostic criteria for idiopathic PD. All PD patients were mid-stage according to the Hoehn-Yahr Parkinson's Rating Scale with a mean score of 2.67 (SD = 0.61) with average age of 64.1 years (SD = 12.1 years). None of the patients were demented according to DSM-IV criteria, clinical judgment, and mean Mini Mental Status Exam (MMSE) scores (mean = 27.0, SD = 1.2). All were on some type of dopaminergic medication and tested while on normal dosing schedules (mean Levodopa Dose Equivalents, LDE of 441.4 mg/day, SD = 231.1). Side of initial onset of PD symptoms was noted for each patient ($N =$ 12, RO; $N = 12$, LO). RO *versus* LO PD patients did not differ significantly in age ($p = 0.50$) years of education ($p = 0.34$), MMSE scores ($p = 0.83$), LDE [$p =$ 0.79], and Unified Parkinson's Disease Rating Scale (UPDRS) scores ($p = 0.70$). Self-reported religious affiliations were 12 Catholics, 5 un-affiliated, 4 Protestant Christians, 1 Jewish, 1 Unitarian, and 1 Jehovah's Witness.

#### *2.1.2. Control participants*

We recruited 24 adult participants from the greater Boston community (8 females) with the average age of 60.9 years (SD = 8.4) and an average of 15.2 years of education (SD = 2.1). Exclusion criteria included history of substance abuse, neurological disorders, major psychiatric diagnoses, or history of brain trauma. After administration of the MMSE, all participants were deemed cognitively unimpaired (mean = 28.5, SD = 1.2). Participants reported a wide range of religious affiliations including 7 Catholics, 6 Protestant Christian, 5 Jewish, 3 un-affiliated, 1 Unitarian, 1 agnostic, and 1 Greek Orthodox.

### *2.2. Prime stories*

We adapted and elaborated on a story-priming procedure from Norenzayan and Hansen (2006) and Cahill et al. (1996) [12,22]. Four similar length- and complexity- matched stories were prepared to represent the following four conditions: mortality salience (Death Prime), encounter with profound beauty in nature (Aesthetic Prime), religious ritual practice (Religious Ritual Prime), and a religiously neutral narration (Control Prime). Each story was 12–15 sentences in length and constructed around a base-narrative – a mother and child leaving home in the morning and visiting the father at work at a hospital. The salient events of the Death, Aesthetic, Religious Ritual and Control prime were the child's untimely death in an accident, the child appreciating an awe-inspiring view of the ocean from atop the hospital, the child observing a prayer ritual in the hospital chapel, and the child watching the hospital staff practice a drill, respectively.

All prime conditions were administered separately to each participant (4 condition repeated-measure) over the course of two test sessions separated by two weeks. To minimize the potential impact on religiosity ratings due to successive readings of similar stories, subjects were randomly assigned to prime condition for two stories separated by two hours in each interview. Order of prime condition presentation was randomized across all participants. An interviewer, blind to the purpose of the study, read a given prime story aloud to the participant while the participant followed along with a printed version of the story. Ten-second pause was given between sentences in order to give participants ample time to imagine details of the story. To assess accurate encoding and reception of the salient events in each prime condition, participants were asked to summarize the story aloud to the interviewer. Responses were transcribed and scored by an independent college-educated rater blind to the purposes of the study. A score of 2 was given if the salient prime event was directly mentioned during story recall, 1 point if the salient prime event was indirectly spoken about, and 0 points if the participant did not recall any details relevant to the prime condition (individual score range 0-8). All subjects completed the Temperament and Character Inventory (TCI) [23]. The TCI sub-dimension *Self-Transcendence* was used as a baseline of belief and covariate in statistical analysis. As a measure of frontal lobe-mediated executive function, age-adjusted Stroop color-word interference scores served as a covariate for belief scores.

### 2.3. Measurement of belief in supernatural agency

After recall of the prime was assessed, each participant was introduced to a brief article from *The New York Times*. Four distinct articles were utilized, however, each was length and complexity matched in addition to including virtually similar themes – religion, prayer, and health. Matching of *The New York Times* articles with prime condition was randomized across all individuals and trials. The experimenter read the article aloud to the participant while the participant followed along with a printed version. Next, participants were given a 12-item survey, which asked subjects their opinion regarding numerous facets of the article. Five of the questions comprised the dependent variable *Belief*, which assessed one's level of belief in God *and* in God as an active agent in the world (using a Likert 9-point scale, 1 = strongly disagree, 9 = strongly agree). The remaining 7-items served as distractions so as not to arouse suspicion in the participants regarding the research aims.

### 2.4. Statistical analysis and transformations

We analyzed the Belief measure as a composite score of the 5 Likert-style questions that addressed belief in God and in God's supernatural agency in the world (Cronbach's alpha = 0.86). Scores on a closed-end scale, such as a Likert scale, cannot be normally distributed because the distribution of ratings for response alternatives falling near either end of the scale must be skewed. The composite data resulted in a negatively skewed data distribution (−0.565). The 9-point Likert scale used in each of the five Belief rating questions across the 4 condition types (Death, Aesthetic, Religious Ritual, and Control) resulted in a twenty number data matrix for each participant. Each individual data matrix was Z-transformed to address the negative skew in the data. Condition distances based on the differences between the Z scores corresponding to each individual and the Z scores derived from the mean reference pattern were used for the following data analysis. Higher Belief scores are interpreted as greater belief in God and God's agency in the world. Likewise, higher Z-scores corresponded with higher Belief scores. Z-transformation of belief scores represents a more rigorous method, given the fact that closed-end Likert scale scores tend not to generate Gaussian response distributions. Without Z-transformation, 'straight-ticket' responders (although very few in our study) across conditions skew the results distribution to favor type I errors if only mean comparisons across condition type are used in statistical analysis.

### 2.5. Patient testing

All patients were tested on two occasions separated by two weeks, and each interview was completed in the morning approximately 1 hour after taking dopaminergic medication. The UPDRS was used to assess severity of PD motor symptoms. TCI Self-Transcendence totals, Stroop age-adjusted color-word interference t-scores, levodopa dose equivalents (LDE), and UPDRS scores were used as covariates in statistical analysis of alterations in Belief Z-scores across prime condition. Z-transformation was used because the data was negatively skewed (−0.735). We covaried for executive function (Stroop Interference t-scores) because previous data implicates frontal function in religious cognition.

PD and control populations did not differ significantly in age, gender, years of education, MMSE scores ($p$s $> 0.2$), or mean post-prime recall test scores (PD mean

= 7.04, SD = 1.27, Control mean = 7.42 out of 8, SD = 0.78; $p = 0.226$). PD patients were tested individually in their homes in the morning hours while on normal medication schedules and controls were tested in a quiet office setting in the outpatient neurology clinic at the Boston VA Hospital. All participants received a small financial honorarium for completion of the study. Research protocols for use in human subjects were approved by Institutional Review Boards from the Boston University School of Medicine and the Department of Veterans Affairs in Boston, MA, USA

### 2.6. Levodopa dose equivalents calculation

We used daily levodopa dose equivalents (LDE) in statistical analyses as a covariate for PD patient Belief Z-scores. LDE was calculated as follows:

Total LDE =
Regular levodopa dose
+ Levodopa CR dose * 0.75
+ pramipexole dose * 67
+ ropinirole dose * 16.67
+ pergolide dose * 100
+ bromocriptine dose * 10
+ if taking COMT inhibitor, then: levodopa dose
+ (CR levodopa dose * 0.75) * 0.25

## 3. Results

### 3.1. Control subjects

Belief Z-scores were highest after exposure to the Aesthetic prime ($z = 0.103$, SD = 0.352) compared to the Death, Religious Ritual, and Control prime Belief scores ($z = -0.050$, SD = 0.557; $z = -0.073$, SD = 0.291; $z = -0.104$, SD = 0.266, respectively) Fig. 1 demonstrates the impact of prime condition on Belief Z-Scores in control participants. Belief Z-scores were used from each of the 4 prime conditions in a repeated-measure ANOVA. The main effect of condition type as a within-subject variable was significant [F (3, 24) = 5.414, $p = 0.002$]. Pair-wise comparisons demonstrated that Belief scores were greater in the Aesthetic versus Control prime condition ($p < 0.05$, $d = 0.66$) and approaching significance in the Aesthetic versus Death prime condition ($p = 0.07$). Religious Ritual and Death prime conditions did not elicit significantly different belief scores from the Control condition ($p = 0.66$ and $p = 0.73$, respectively).

Both of the covariates, TCI Self-Transcendence scores and executive function (Stroop) scores covaried with the main effect of the prime condition on Belief scores [F (3, 24) = 2.92 $p = 0.041$, $\eta^2 = 0.122$, power = 0.667; F (3, 24) = 4.18, $p = 0.009$, $\eta^2 = 0.166$, power = 0.833, respectively]. The greater the TCI Self-Transcendence scores and higher Stroop executive function scores covaried with greater prime condition effect on Belief Z-scores. Post-prime memory tests demonstrated efficient recall of salient events by all participants (mean = 7.42 out of 8, SD = 0.78).

### 3.2. PD patients

Belief Z-scores were highest after exposure to the Aesthetic prime ($z = 0.172$, SD = 0.380) compared to the Death, Religious Ritual, and Neutral prime Belief scores ($z = 0.022$, SD = 0.373; $z = -0.067$, SD = 0.411; $z = -0.119$, SD = 0.398, respectively). Belief Z-scores were used from each of the 4 prime conditions in a repeated-measure ANOVA. The main effect of condition type as a within-subject variable was significant [F (3, 24) = 4.039, $p = 0.05$]. Pair-wise comparisons demonstrated that Belief scores were greater in the Aesthetic versus Control prime condition ($p < 0.05$). Religious Ritual and Death prime conditions did not elicit significantly different belief scores from the baseline neutral condition ($p = 0.32$ and $p = 0.67$, respectively).

To compare the impact of prime condition and group (PD versus control) on Belief Z-scores a 2 (group) × 4 (prime condition) ANOVA was performed. The main effect of prime condition was significant ($F = 5.529$, $p < 0.001$) for both PD and control groups. The main effect of group or group by prime condition interactions was not significant (ps > 0.7). When PD subjects were divided by side of disease onset (LO and RO) and entered into a 2 (group) × 4 (prime condition) ANOVA, the main effect of prime condition was significant ($F = 3.344$, $p < 0.02$) in addition to the side of onset group by prime condition interaction effect ($F = 2.671$, $p < 0.05$).

To assess the impact of PD laterality and executive function on Belief, we used the four-condition Belief Z-scores as dependent variables in a repeated-measure ANOVA with side of PD onset as a between-subject variable, and UPDRS scores, LDE, Stroop age-adjusted color-word interference t-scores, and TCI Self-Transcendence scores served as covariates. Side of onset exhibited significant impact on Belief scores, most evident in the Aesthetic prime condition [F (3,

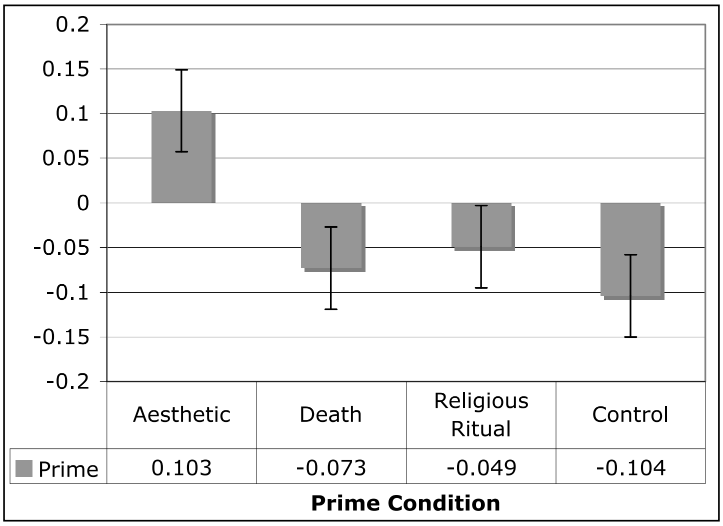

Fig. 1. Z-transformed Belief Scores from 24 neurologically healthy aged-adults following exposure each to 4 prime conditions: Aesthetic, Death, Religious Ritual, a non-religious Control priming. Note: Error bars represent standard error of the mean. Religious Belief increased significantly following exposure to Aesthetic priming compared to control conditions, and Belief decreased slightly following Death priming.

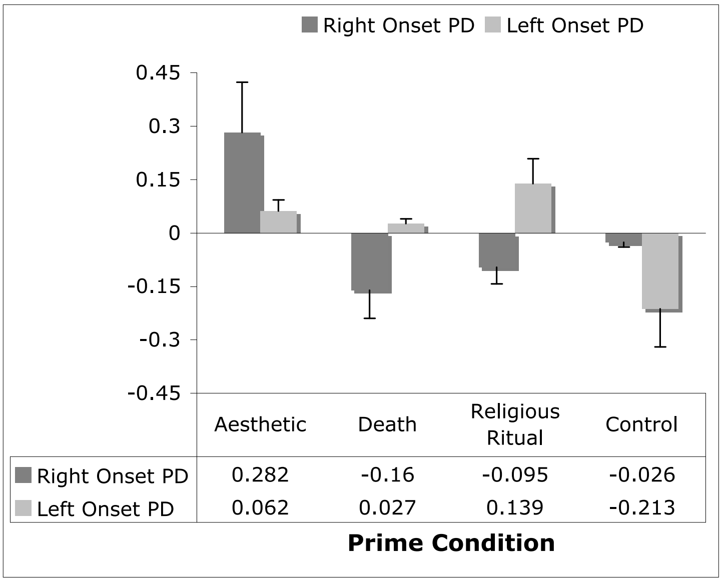

Fig. 2. Z-transformed Belief Scores from 24 adult mid-stage PD patients following exposure each to 4 prime conditions: Aesthetic, Death, Religious Ritual, and a non-religious Control priming. Note: Error bars represent standard error of the mean. Change in Belief Z-scores is displayed as a function of disease onset with right-onset PD indicating greater left forebrain disease and vice versa.

24) = 7.946, $p$ = 0.022]. Figure 2 demonstrates the impact of disease laterality on Belief Z-Scores based on prime condition. Following the Aesthetic prime condition, RO PD patients' experienced significant increases in Belief, whereas, LO subjects Belief scores were stagnate and no different than Control baseline scores ($z$ = 0.282, SD = 0.371 and z = 0.062, SD = 0.371, respectively, $p$ = 0.11). LO PD subjects exhibited the following belief scores for Aesthetic, Death, Religious Ritual, and Control prime conditions, respectively: 0.06 (SD = 0.37), 0.03 (SD = 0.43), 0.14 (SD = 0.34), and −0.21 (0.40). Only increased Belief following the Religious Ritual versus Control prime conditions under planned comparisons achieved significance ($p$ = 0.029). Aesthetic and Death prime belief scores did not differ in LO PD subjects. In RO PD patients, Belief scores for

Aesthetic, Death, Religious Ritual, and Control prime conditions were: 0.28 (SD = 0.37), −0.16 (SD = 0.39), −0.10 (SD = 0.38), and −0.03 (SD = 0.39), respectively. Comparisons achieving significance include greater Belief in Aesthetic versus Death prime condition ($p = 0.009$, $d = 0.82$) and Aesthetic versus Religious Ritual condition ($p = 0.02$).

Of the covariates, only Stroop scores of executive function (inhibition and task switching) covaried with change in Belief scores [F (3, 24) = 4.505, $p = 0.019$, $\eta^2 = 0.474$, power = 0.779]. Greater executive function as measured by Stroop covaried with greater increase in Belief Z-score by condition.

## 4. Discussion

We investigated whether modulation of religiosity via priming techniques (mortality salience, religious ritual, and aesthetic) would differ in patients with PD as compared to neurologically intact controls. We found that modulation of religiosity levels among PD patients was reduced relative to the controls. We also examined the impact of modulation of religiosity within the PD group as a function of side of onset of disease. Compared to both controls and right onset (RO; implying left forebrain dysfunction) PD patients, left-onset (LO; implying right forebrain dysfunction) patients did not exhibit an increase in religious belief following the Aesthetic prime condition. On the other hand the religious-ritual prime did significantly increase belief for LO PD patients. Interestingly, RO PD patients manifested a 'hyper-priming effect' with a dramatic increase in belief following the Aesthetic prime compared to LO patients and controls (Cohen effect size of RO PD *versus* controls, 0.82 and 0.66, respectively). These results correlated with measures of frontal executive function in both PD patients and controls. The double-dissociation in ritual compared to aesthetic religious cognition in RO versus LO patients suggests that right frontal dopaminergic tone is important for aesthetic-mediated religiosity and left frontal is key to ritual-based religious cognition.

We will discuss the theoretical significance of the results we obtained from the neurologically intact controls before discussing the significance of the double-dissociation we found in the PD patient groups. The finding from neurologically intact age-matched adults is noteworthy. Using a repeated-measure analysis, individual differences in Belief scores significantly increased following exposure to an Aesthetic prime compared to the non-religious Control baseline condition. Important to note, we did not replicate the findings of Norenzayan and Hansen (2006) who reported that mortality salience significantly increased belief in supernatural agency. In fact, Belief scores in our healthy controls *decreased* following the Death prime compared to Aesthetic prime. The failure to replicate Norenzayan and Hansen's results may be due to several methodologic differences between the two studies. We improved on the Norenzayan and Hansen design by using a within-Ss repeated-measure design methodology and we eliminated statistical departures from normality by using Z-transformed Belief scores. Further, our study population was aged- and not young- adults, and religiously diverse rather than predominately Christian. Beyond these methodologic improvements over the Norenzayan and Hansen study we believe that it is reasonable to suppose, along with generations of scholars who have studied religious experiences, that religious experiences are driven primarily by love of beauty, truth and other ultimate values rather than by fear of death as Norenzayan and Hansen and some other theorists propose. When given a choice even elderly people (who presumably are closer to death than the young people in Norenzayan and Hansen studies) enhance their religious responding preferentially to the aesthetic rather than to the fear of death condition. Our data do not support theories of religiosity represented as security blanket for fearful, deluded people not able to handle thoughts of death.

We also obtained a double dissociation in neuropsychological data. RO patients exhibited a significant increase in belief following the Aesthetic prime condition but not the ritual prime and LO patients significantly increased belief in the religious ritual prime condition but not the aesthetic prime condition. These performance patterns, furthermore, correlated with performance on executive function tasks. We interpret this set of findings to suggest lateral cerebral specialization for ritual-related (left frontal) versus aesthetically-driven (right frontal) religious cognition. Of course these results will need to be replicated by further work before they can be taken as definitive but we nevertheless find them consistent with what little is known about the brain basis of religious cognition more generally. For example, aesthetically linked religious cognition is likely linked to forms of religious experience that involve appreciation of beauty, the sublime and altered states of consciousness more generally. A whole range of neuropsychologic data (see review in McNamara, 2009, ref. 10) from an examination of clinical disorders involving enhancements in religiosity after brain stimulation or ingestion of entheogenic drugs suggests that right prefrontal and medial temporal networks consistently mediate these sorts of religious responses. Conversely, religious ritual form has been analyzed in linguistic terms as sets of prescribed sequential actions that refer to supernatural agents. To the extent that ritual form overlaps with language structure and programming of behavioral actions more generally it seems reasonable to us to suppose that left hemisphere language related neural networks would preferentially mediate ritual forms of cognition. Thus, although we did not predict the double dissociation that we in fact obtained we find it a reasonable set of findings given what we know about brain correlates of both aesthetic and ritual cognitions.

The fact that religious responding was correlated with performance on executive function tests is also consistent with McNamara's theory (McNamara, 2009) concerning the functions of religious cognition vis-à-vis religion's role in self-regulation [10]. Religious cognitions and practices are theorized to facilitate recruitment of executive control processes in the frontal lobes to better regulate behavioral strategies aimed at achieving long term goals rather than short term rewards. If religion does facilitate self-regulation in service to long term goals then it is not surprising to find that religiosity responses vary with performance on executive function tests.

Our identification of alterations in religiosity in patients with PD is consistent with the evidence from recent neuroimaging and clinical studies, which implicates dopaminergic and serotonergic activity within frontotemporal brain networks in support of religious cognition [11,31–38]. Given that dopaminergic activity is reduced in PD in frontotemporal networks it is not surprising that overall responding to religious primes is reduced in PD patients relative to healthy controls. But again we feel that our most interesting set of results is the dissociation in religious responding within the PD group based on side of onset and prime type. Our results imply that differing religious coping strategies will be differentially available to distinct neurobehavioral phenotypes based on side of onset. For LO patients ritual forms of religious cognition will be easier (because they will have an intact left prefrontal network) than the 'altered states of consciousness' forms of religious cognition for RO patients (who will have an intact right prefrontal network to work with) and vice versa. Relative to healthy control all forms of religious cognition will be more difficult to access for PD patients than controls.

Participation in religious activity is important to many aged individuals, including patients with PD and correlates with quality of life and positive health outcomes [39–54]. For clinicians managing complex, individualized care for patients with chronic disease, it is imperative to know the role religiosity plays in health outcomes, cognitive decline, quality of life, decisions regarding palliative care, neuropsychiatric health, and patient compliance.

## Acknowledgements

The authors report no conflicts of interest. This is based on work supported by the Office of Research Development, Medical Research Service, Department of Veterans Affairs and the National Institute of Deafness and other Communication Disorders (NIDCD) Grant No. 5RO1DC007956-03.
